# Construction of a gamma-retrovirus with a novel tropism dependent on human APJ as entry receptor

**DOI:** 10.1186/1742-4690-8-S2-P41

**Published:** 2011-10-03

**Authors:** Shervin Bahrami, Kristina PS Kristensen, Ditte E Mortensen, Mogens Duch, Martin Tolstrup, Finn S Pedersen

**Affiliations:** 1 Department of Molecular Biology and Genetics, Aarhus University, DK-8000 Aarhus, Denmark; 2 Enfutech, 8200 Aarhus N, Denmark; 3 Department of Infectious Diseases, Aarhus University Hospital, DK-8200 Aarhus N, Denmark

## Background

The gamma-retrovirus isolate SL3-2 belongs to the group of xenotropic-polytropicmurine leukemia viruses which uses the xenotropic-polytropic receptor Xpr1 for entry[1]. However, the SL3-2 isolate has a tropism restricted to murine cells, which makes SL3-2 interesting for receptor-retargeting experiments.

To investigate SL3-2 as a new scaffold for retargeting experiments an alternativeentry receptor has been chosen, the human G-protein coupled receptor hAPJ. In addition, hAPJis known to be able to function as a co-receptor for some HIV-1 isolates.

## Methods and materials

The chimeric envelope protein was made by insertion of the natural ligand of hAPJ, apelin, flanked by linker sequences[2], into one of the variable loops of the receptor binding domain of the SL3-2Env. This is possible due to the small size of the 13 amino acids long apelinpeptide. The tropism of the resulting envelope protein was determined using a retroviral vector system. Furthermore, a replication competent retrovirus was made by replacing the envelope protein of SL3-3 MLVwith the chimeric envelope protein of SL3-2(SL3-AP).

## Results

The SL3-AP envelope protein can use hAPJ as well as its natural receptor murine Xpr1 for entry into host cells with equal efficiencies. The SL3-AP virus can replicate in cells expressing either of its receptors: hAPJ or murine Xpr1 and shows down regulation of hAPJ in infected cells.

## Discussion

The SL3-AP is the first example of a retargeted replication competent retrovirus, with replication characteristics similar to natural isolates. Furthermore, it has been possible to maintain the viral infectivity through more than four passages in canine-hAPJ cells. The tropism of the virions from the later passages as well as the sequence of their envelope genes will be determined in order to establish any possible evolution of the retargeted envelope protein under selective pressure for infection through hAPJ.
